# Copper catalysis with redox-active ligands

**DOI:** 10.3762/bjoc.16.77

**Published:** 2020-04-24

**Authors:** Agnideep Das, Yufeng Ren, Cheriehan Hessin, Marine Desage-El Murr

**Affiliations:** 1 Université de Strasbourg, Institut de Chimie, UMR CNRS 7177, 67000 Strasbourg, France; 2 Sorbonne Université, Institut Parisien de Chimie Moléculaire, UMR CNRS 8232, 75005 Paris, France

**Keywords:** bioinspired catalysis, biomimetic copper complexes, cooperative catalysis, redox-active ligands, redox catalysis

## Abstract

Copper catalysis finds applications in various synthetic fields by utilizing the ability of copper to sustain mono- and bielectronic elementary steps. Further to the development of well-defined copper complexes with classical ligands such as phosphines and N-heterocyclic carbenes, a new and fast-expanding area of research is exploring the possibility of a complementing metal-centered reactivity with electronic participation by the coordination sphere. To achieve this electronic flexibility, redox-active ligands can be used to engage in a fruitful “electronic dialogue” with the metal center, and provide additional venues for electron transfer. This review aims to present the latest results in the area of copper-based cooperative catalysis with redox-active ligands.

## Introduction

Interaction of earth-abundant metals, such as copper, with radical ligands is originally known from biological systems such as metalloenzymes [1]. Among the myriad of existing enzymes, galactose oxidase (GAO) is a copper-based enzyme performing the two-electron oxidation of galactose through a mechanism involving the metal and a neighboring tyrosine radical ligand for the shuttling of overall two protons and two electrons. This peculiar mechanism is enabled by transient storage of electronic density on the tyrosine ligands and perfectly illustrates the central role of ancillary pro-radical ligands in biological systems. Among other tasks, copper enzymes are known to be actively involved in electron transfer as exemplified by blue copper enzymes, which have captured the interest of chemists and biochemists. Copper can also cooperate with iron to perform activation of O_2_ and nitrogen oxides (NO*_x_*) in cytochrome c oxidases [2–3]. Inspired by the broad chemical repertoire of radical-ligand containing metalloenzymes, chemists have developed redox-active ligands as surrogates for the biologically occurring redox cofactors and this has translated into active developments in homogeneous catalysis [4]. The unique electronic interaction due to matching or inverted energy levels between metal and ligand leads to valence tautomerism [5–8], which paves the way for orchestrated electronic events occurring within the metal complex and influences the chemical reactivity of the complex. While the field of catalysis with redox-active ligands is itself a much broader area, we shall limit our discussion to copper catalysis with redox-active ligands and the present review aims at providing an overview of the relevant literature on this topic over the last three decades.

## Review

### Oxidation: C–O bond formation

The central role of copper intermediates in oxidation reactions in biological systems is very well documented [9]. It is therefore not surprising that this would be an area of choice for the development of bioinspired and/or redox-active copper complexes.

**Scheme 1:**
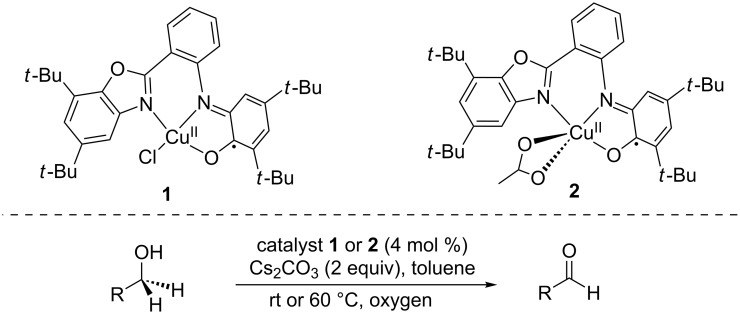
Copper complexes with amidophenolate type benzoxazole ligands for alcohol oxidations.

Inspired by the pioneering works by Stack [10], Wieghardt and Chaudhuri on GAO mimics [11–13], Safaei and Storr have reported Cu(II) complexes bearing a non-innocent amidophenolate type benzoxazole ligand which was fully characterized by X-ray crystallography and other spectroscopic techniques (Scheme 1) [14]. These complexes were found to perform the aerobic catalytic oxidation of alcohols into aldehydes, thus mimicking the reactivity of galactose oxidase. Complexes **1** and **2**, exhibiting a twisted tetracoordinated geometry, and a distorted square pyramidal geometry, respectively, were used in alcohol oxidation on a wide variety of mostly aromatic alcohols in the presence of base (Cs_2_CO_3_). The results suggested that **2** is more efficient than **1** and this reactivity difference could be attributed to an increased stabilization of the involved reactive catalytic intermediates by the electron-donating acetate ions present in complex **2**.

**Scheme 2:**
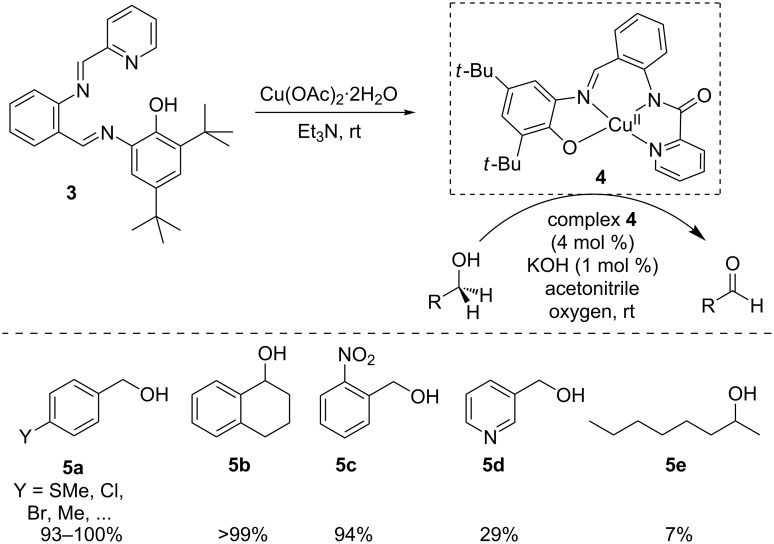
Copper-catalyzed aerobic oxidation of alcohols and representative substrate scope.

The same team later reported on another family of galactose oxidase mimics based on copper(II) complex **4** bearing a non-innocent iminophenol-iminopyridine hybrid ligand **3** that performed two-electron oxidations of primary alcohols to aldehydes (Scheme 2) [15]. Catalyst **4** was fully characterized by combined EPR, cyclic voltammetry and electronic spectroscopic studies to reveal two quasi-reversible one-electron redox processes, including a ligand-centered oxidation generating a Cu^II^-phenoxyl radical species, thus suggesting the electronic participation of the ligand in the redox processes. This catalyst is efficient in the oxidation of a large number of alcohols (**5a–e**) in the presence of base (KOH 1 mol %).

**Scheme 3:**
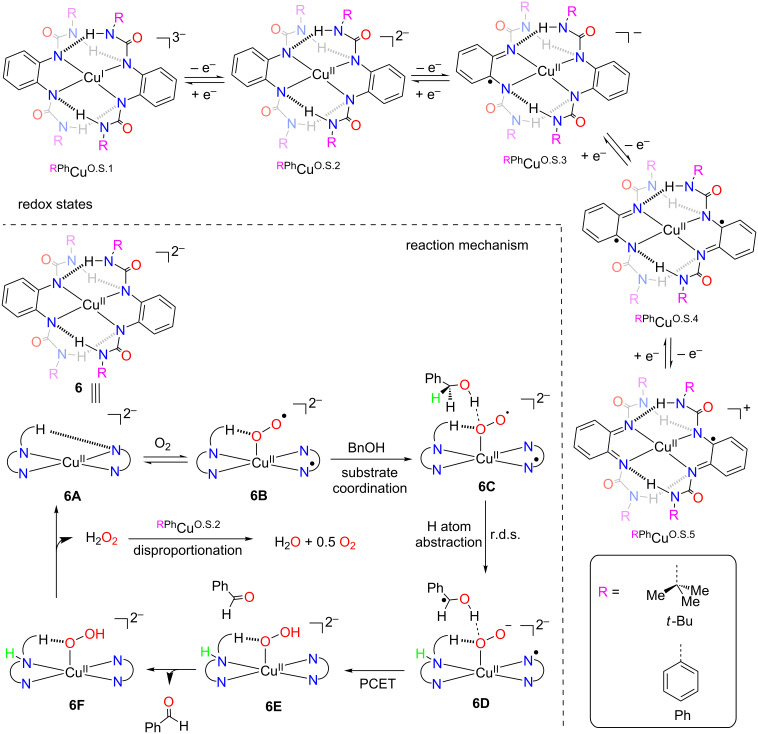
Introduction of H-bonding network in the ligand coordination sphere.

An interesting strategy aiming to explore the possibility of interfacing the redox-activity with H-bonding ability inside the ligand coordination sphere has been disclosed by Swart and Garcia-Bosch [16], and relies on the design and study of a new copper(II) complex. A family of *o*-phenylenediamido ligands was synthesized through the introduction of ureanyl groups, using a synthetic approach developed by Borovik [17–18] for the stabilization of metal–oxo and metal–hydroxo complexes via intramolecular H-bonding interactions. The resulting copper complexes were synthesized and single crystal X-ray diffraction analysis evidenced the existence of H-bonding inside the coordination sphere of **6** (Scheme 3). The authors reported that the influence of the geometry of the complex on the H-bonding interactions as well as the nature of this interaction greatly affected the chemical properties. They also reported that complexes that had higher oxidation potential and went through irreversible oxidation were not good catalysts for the oxidation of benzyl alcohol to benzaldehyde (0–30% yields, e.g., ^PhPh^Cu^O.S.2^, O.S.*x* signifies oxidation state and *x* increases with the removal of electrons). On the other hand, complex *^t^*^-BuPh^Cu^O.S.2^, showing a reversible oxidation behavior very efficiently performs dehydrogenation of benzyl alcohol with low catalyst loading (0.1 mol %) and high turnover numbers (TONs up to 184). Besides the oxidation of benzyl alcohol to benzaldehyde, the authors also show that reaction with ^t-BuPh^Cu^O.S.2^ (5 mol %) could be efficiently extended to other substrates containing weak C–H bonds and acidic O–H bonds such as diphenylmethanol (PhBzOH), benzoin, and cinnamyl alcohol. However, alcohols with stronger C–H bonds (cyclohexanol) and more basic O–H bonds (1-phenylethanol, MeBzOH ) were either not oxidized at all, or in very low yields.

Relying on a combination of kinetic and computational studies, the authors propose a detailed mechanistic overview (Scheme 3) for the transformation. A proposed model mechanism involves reaction of Cu^II^ complex **6A** with dioxygen to generate a Cu^II^ superoxide intermediate **6B**. The electron needed to reduce O_2_ to superoxide is provided by the redox-active ligand. Subsequent coordination of benzyl alcohol results in the formation of species **6C**, which undergoes a H-atom abstraction step to form **6D**, in which the H-atom is transferred from the secondary benzylic sp^3^ carbon to the redox-active ligand, acting as a cooperative H-atom acceptor. Following a proton-coupled electron transfer (PCET) to generate **6E**, the oxidized product (benzaldehyde) is released and final elimination of H_2_O_2_ regenerates the active catalyst, thus closing the catalytic cycle. Interestingly, this catalytic behavior involving participation of the ligand framework to release electrons and store an H-atom is reminiscent of the galactose oxidase copper enzyme.

**Scheme 4:**
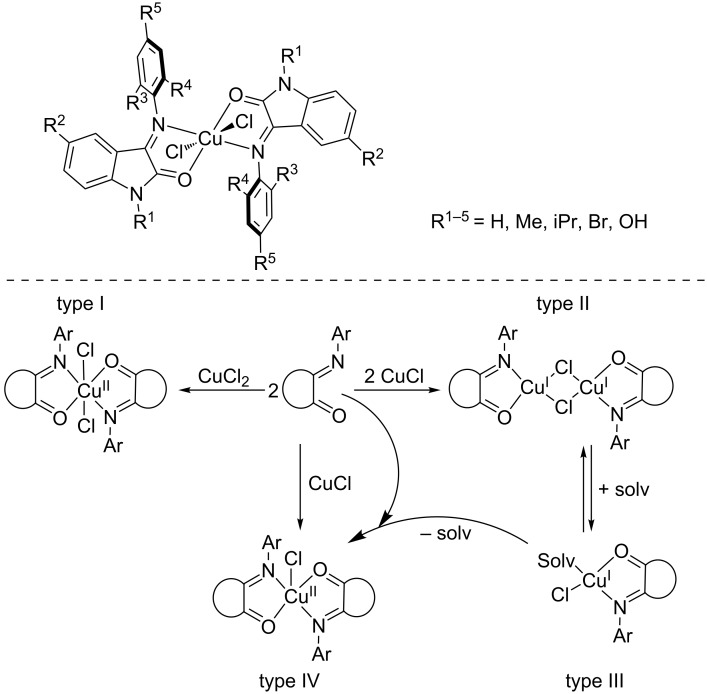
Well-defined isatin copper complexes.

A recent investigation by Martins, Davidovich and co-workers reports on copper complexes of the redox-active Schiff-base isatin bearing various functional groups (Scheme 4) [19]. The main feature of these ligands is their ability to form four distinct and well-defined types of complexes depending on the reaction conditions (i.e., the number of equivalents of metal precursor, solvent, etc.). These complexes performed the oxidation of benzyl alcohol into benzaldehyde and the results suggested that the optimization of the ligand structures by the introduction of different substitutions leads to better catalytic efficiency. The redox behavior of the ligands and complexes was investigated through electrochemical studies and suggested to proceed via an electrochemical chemical event (EC) including reversible electrochemical and irreversible chemical steps. The authors also observed a diminished stability of the complexes upon reduction, and this low redox stability could be circumvented through ligand modification.

**Scheme 5:**
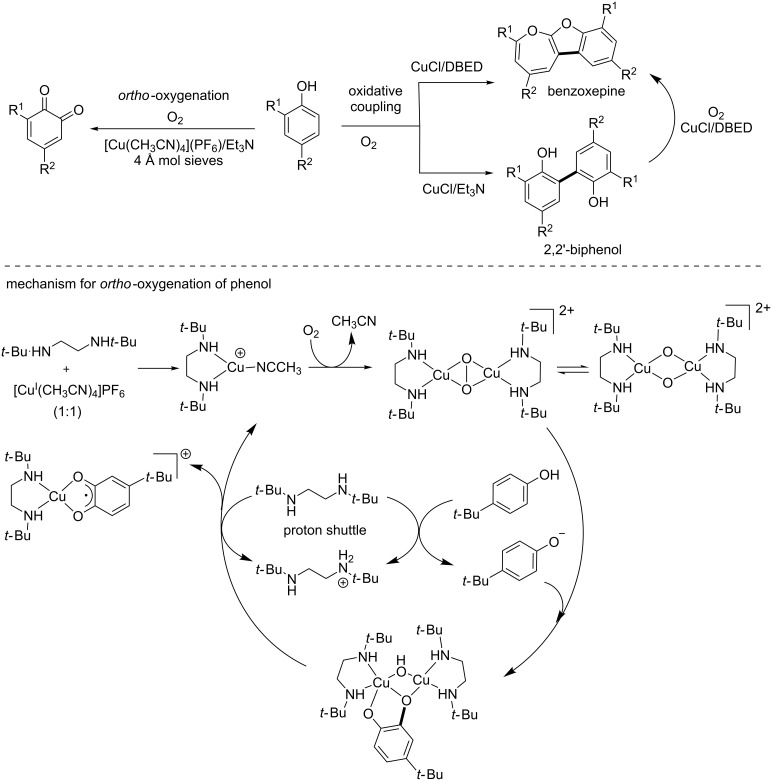
Catalyst control in the biomimetic phenol *ortho*-oxidation.

Phenol oxidation is ubiquitous in biological systems as demonstrated by the involvement of the copper enzymes tyrosinases (type III) in the melanogenesis process. The regioselectivity and reactivity of the oxidation of phenols are strongly dependent on the phenol substrate substitution pattern, and it is therefore difficult to develop general methods for this transformation. This substrate–control bias was successfully outmaneuvered by the development of a copper-based catalytic system operating under aerobic conditions and allowing selective access to benzoxepines and 2,2’-biphenols through catalyst control (Scheme 5, top) [20].

**Scheme 6:**
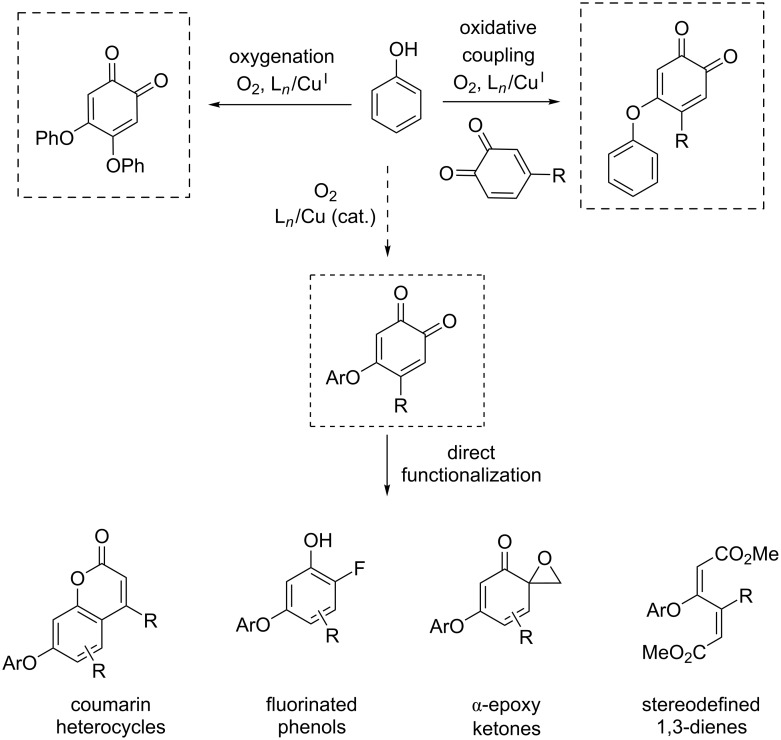
Structural diversity accessible by direct functionalization.

Extensive mechanistic studies evidenced a biomimetic pathway involving a key Cu^II^-semiquinone intermediate in which the substrate becomes activated as a redox-active ligand (Scheme 5, bottom) [21]. This radical Cu^II^ species is the catalyst resting state and is accessed starting from a dinuclear side-on peroxodicopper(II) intermediate, thus closely mimicking copper tyrosinases [22–23]. Interestingly, further studies have focused on a closely related combination of copper salt, diamine (DBED: *N*,*N*’-di-*tert*-butylethylenediamine) for the oxidation of benzyl alcohol to benzaldehyde and shown that under aerobic conditions, the system leads to the formation of a nitroxyl radical on the DBED moiety [24]. This newly formed radical acts as a redox-active cocatalyst in the oxidation reaction, which has previously been reported with TEMPO (2,2,6,6-tetramethyl-1-piperidine *N*-oxyl) [25] and ABNO (9-azabicyclo[3.3.1]nonane *N*-oxyl) radicals [26]. Later reports enlarged the synthetic scope of this methodology and provided access to a wide range of synthetically useful building blocks such as substituted heterocycles, fluorinated phenols, ketones and 1,3-dienes (Scheme 6) [27].

### C–C bond formation

**Scheme 7:**
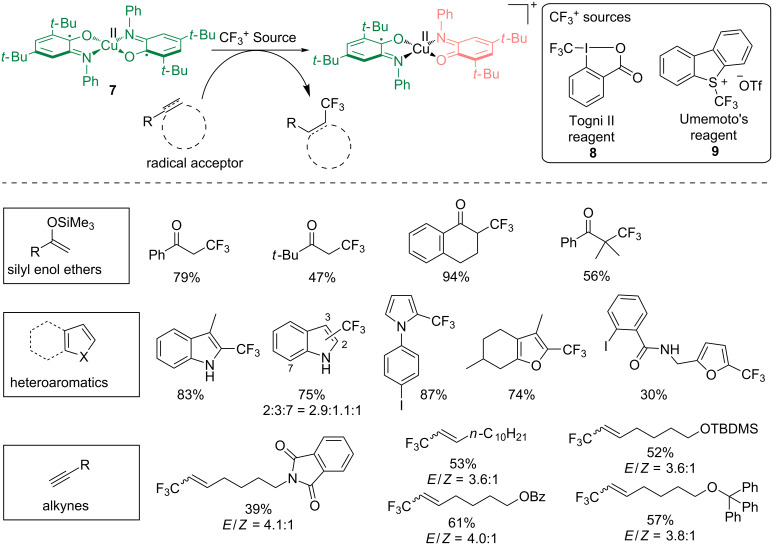
Copper-catalyzed trifluoromethylation of heteroaromatics with redox-active iminosemiquinone ligands.

Complexes of radical and redox-active ligands with transition metals are known to be able to promote radical reactions through single-electron transfer (SET) processes [28]. Expanding on the research area pioneered by Wieghardt and Chaudhuri [11–13] using well-defined copper complex **7**, originally reported as a GAO mimic [20–22], we showed that this ability could be extended to the catalytic generation of trifluoromethyl radicals (Scheme 7) [29]. The synthetic efficiency of complex **7** in trifluoromethylation was reported on a wide variety of substrates including silyl enol ethers, heteroaromatics and alkynes using an electrophilic CF_3_^+^ source (**8** or **9**), opening new opportunities to access pharmaceutically relevant trifluoromethylated products under mild reaction conditions. The ligand-based SET step involved the iminosemiquinone redox-active ligand which was oxidized to iminobenzoquinone.

**Scheme 8:**
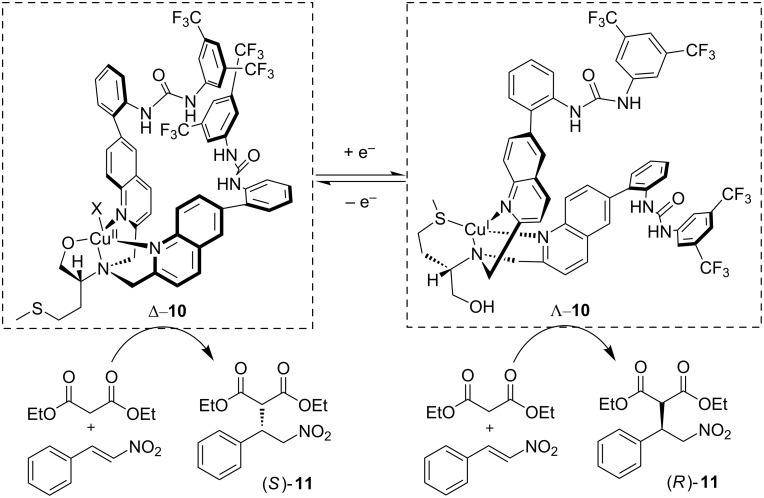
Reversal of helical chirality upon redox stimuli and enantioselective Michael addition with a redox-reconfigurable copper catalyst.

The Canary group [30] reported a redox-reconfigurable copper catalyst that exhibits reversal of its helical chirality through redox stimuli (Scheme 8). Combining ʟ-methionine and catalytic urea groups with two different copper salts as precursors affords both enantiomers Δ-**10** (from CuClO_4_) and Λ-**10** from (Cu(CH_3_CN)_4_PF_6_). UV–vis and circular dichroism spectroscopic studies evidence that the helical chirality exhibited by these two catalysts could be reversed by redox stimuli. These complexes could perform enantioselective Michael addition between diethyl malonate and nitrostyrene to afford only one of the two (*S*)-**11** and (*R*)-**11** enantiomers, depending on the redox state of the copper center. The reaction was run with a 5 mol % catalyst loading and a base (Et_3_N) and was compatible with different solvents (THF, MeCN, CH_2_Cl_2_ and hexane). Acetonitrile gave the highest yield (55%) and ee (72%) of product (*S*)-**11** in presence of Λ-**10** as catalyst. While the free ligand is found to catalyze the reaction, the authors show that the templating effect of the copper ion is necessary to sustain enantioselectivity.

**Scheme 9:**
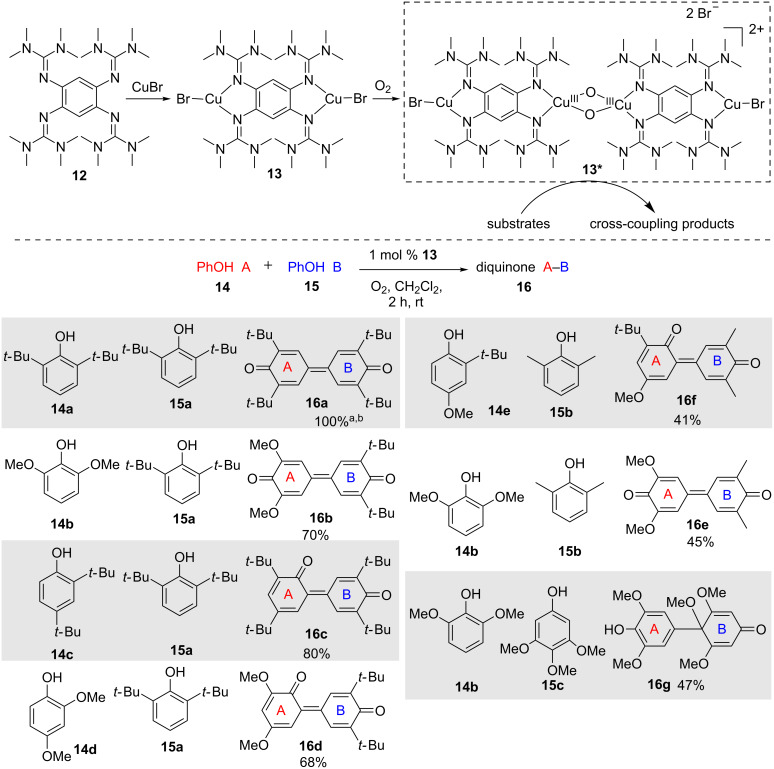
Interaction of guanidine-copper catalyst with oxygen and representative coupling products. ^a^4 mol % catalyst loading, ^b^homo-coupling adduct.

Himmel and co-workers have reported the use of copper(I) complexes bearing redox-active guanidine ligands **12** for catalytic aerobic homo- and cross-coupling of phenols with dioxygen as oxidation source (Scheme 9). This strategy allowed access to nonsymmetrical biphenols and the best results were obtained with dinuclear complex **13** incorporating two copper(I) centers. These specific quinidine ligands have the ability to supply electrons to the metal center thanks to their low oxidation potential and can shuttle up to two electrons to the copper centers. The specific geometry of this ligand also has an influence on the rate of electron transfer through a “structural harmonization” between the Cu^II^ and Cu^I^ redox states through an entatic state geometry [31–32]. According to the authors’ findings, **13** initially reacts with dioxygen to generate an unstable bis-μ-oxo-complex intermediate 13* which is the active species during the catalytic cycle for the C–C coupling. The coupling reaction follows a radical–anion mechanism due to the presence of electron-donating ligand **12** and this is the key feature allowing preferential binding of the phenoxyl radicals for PCET. Products arising from homo- and heterocouplings were formed with high selectivity. Homo- and cross-coupling adducts **16a** and **16b–g** were obtained from the corresponding phenols (**14a–e** and **15a–c**) as shown in the bottom of Scheme 9 with high chemoselectivity.

### C–N bond formation

While copper complexes are at the heart of oxidative biological networks, the introduction of nitrogen is a different matter. The Arnold group has elegantly shown that through iterative mutagenesis “directed evolution” enzymes can be coaxed to perform unnatural transformations such as C–N bond forming aziridination, which has no biological counterpart [33]. While the exact structure of the mutant metalloenzymatic active site was not characterized in detail, it might bear some resemblance to the original biological active site.

**Scheme 10:**
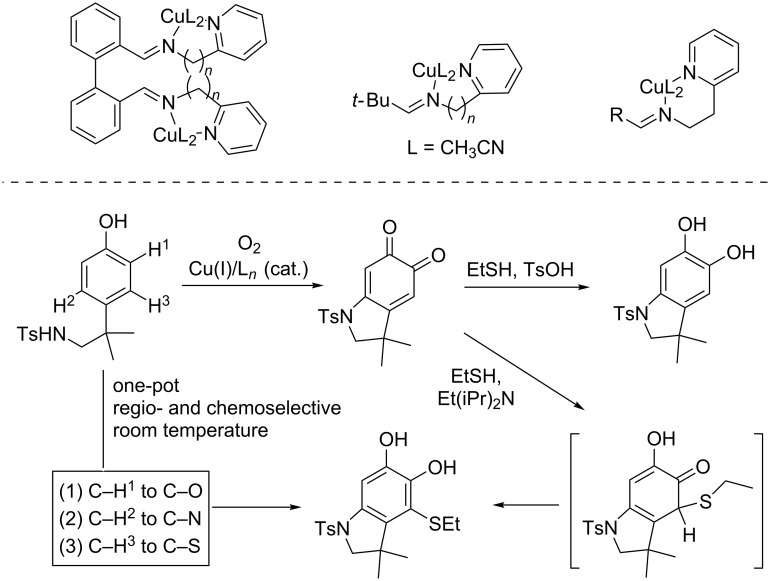
Access to 1,2-oxy-aminoarenes by copper-catalyzed phenol–amine coupling.

Building on their previously discussed phenol oxidation methodology (Scheme 5 and Scheme 6), Lumb and co-workers have targeted subsequent C–N bond formation to access oxindoles [34], and perform C–H functionalization through aerobic dearomatization of phenols [35]. These broad synthetic outcomes further led to a unified approach for the preparation of 1,2-oxy-aminoarenes by phenol–amine couplings (Scheme 10) [36].

**Scheme 11:**
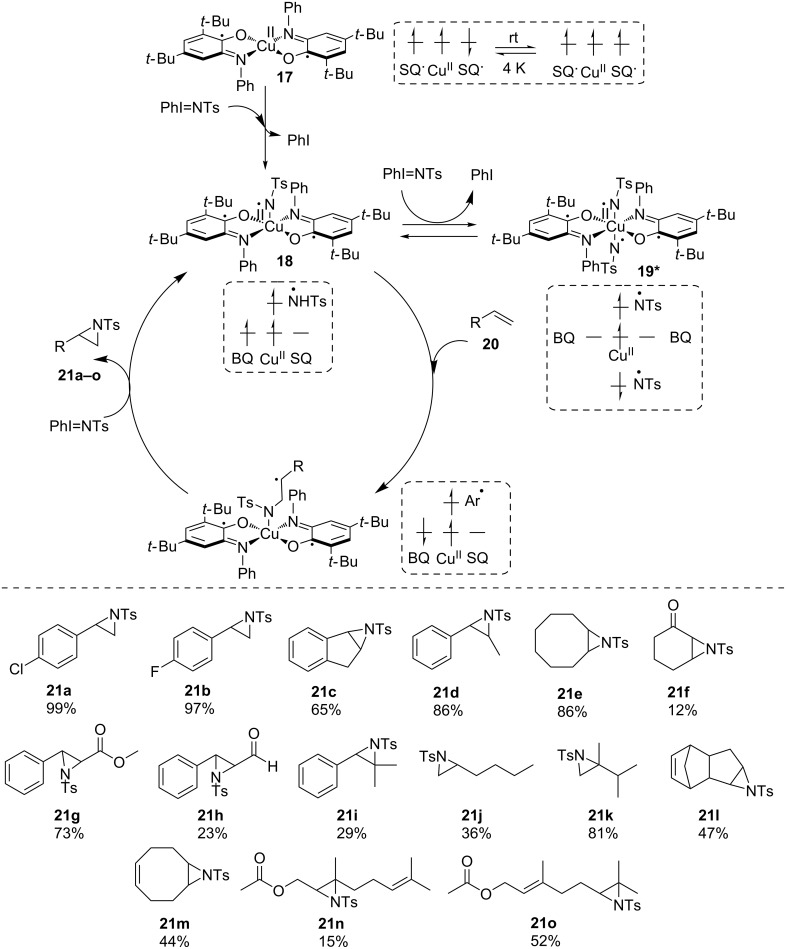
Copper-catalyzed aziridination through molecular spin catalysis with redox-active iminosemiquinone ligands.

We reported the formation of a C–N bond through copper-catalyzed aziridination with redox-active complex Cu(SQ)_2_ **17** [37], originally developed as a GAO mimic. The reaction worked very efficiently under ambient conditions with *N*-tosyliminobenzyliodinane (PhINTs) as a nitrene source. The reaction mechanism was proposed to involve a transient mono-nitrene Cu^II^ intermediate **18** in equilibrium with a spectator bis-nitrene species **19** and proceeded through styrene **20** insertion, followed by ring closure and release of the aziridine. Interestingly, this otherwise classical mechanism is enabled by the accessibility of doublet and quadruplet states close in energy and this molecular spin catalysis is reminiscent of the multistate reactivity observed at the metal center, for example in heme enzymes [38–39]. A wide range of substrates could be converted including mono-substituted aziridines (**21a**, **21b** and **21j**), disubstituted aziridines (**21c–h** and **21l**,**m**), and more challenging scaffolds such as gem-disubstituted (**21k**) and trisubstituted (**21i** and **21n**,**o**) aziridines (Scheme 11). Furthermore, the reaction conditions are compatible with aldehyde, ester or ketone functions.

**Scheme 12:**
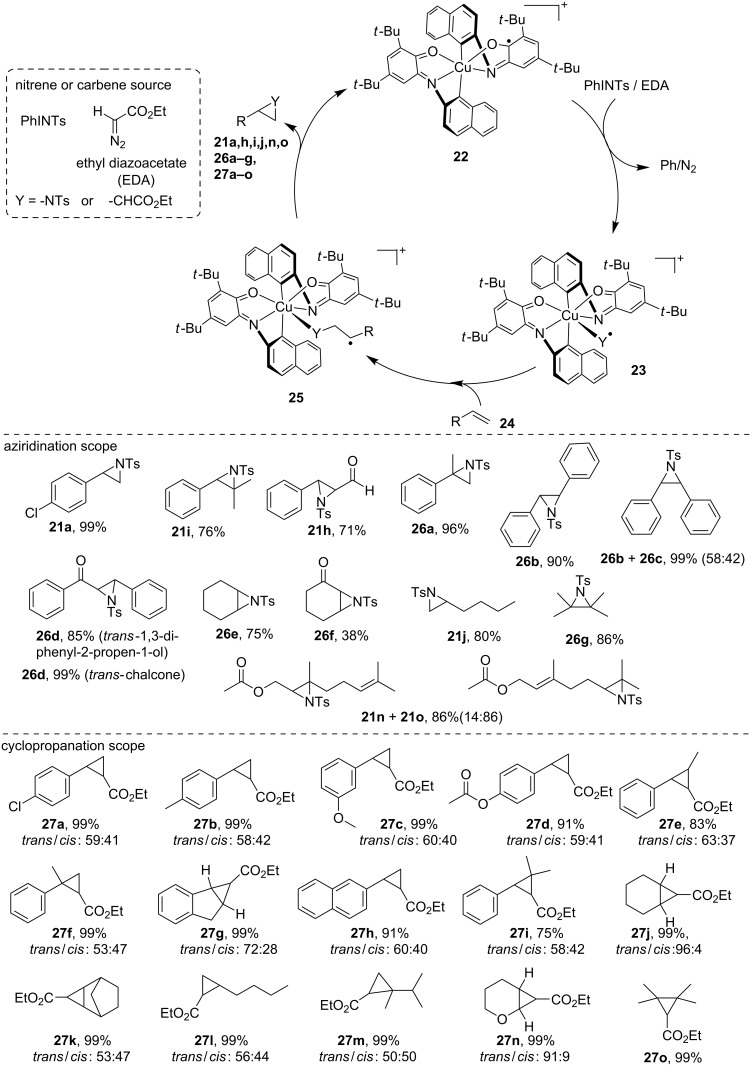
Nitrogen-group and carbon-group transfer in copper-catalyzed aziridination and cyclopropanation through a strained redox-active ligand framework reminiscent of the entatic state model. Products are obtained as a mixture of *cis* and *trans* isomers.

Metalloenzymes routinely rely on 3d metals and amino acid-derived coordination spheres to perform complex (multi)electronic transformations of paramount importance in atom transfer reactions and activation of small molecules. To do so, metalloenzymes have acquired many evolutionary reactivity-enhancing tools that enable efficient chemical processes. Among these tools, the entatic state model relies on the fact that a strong steric distortion induced by ligands in the coordination sphere around a metal center produces an energized and highly reactive structure. Our recently published study revealed, that use of a highly-strained redox-active ligand facilitates a transfer of nitrogen- and carbon-containig group by copper complex **22** in as fast as two minutes, and therefore, it exhibits a strong increase in reactivity when compared to its unstrained analogue [40]. This example of a bioinspired small-molecule synthetic system combines two reactivity-enhancing features from metalloenzymes: entasis and redox cofactors. Strikingly, combination of these unique steric and electronic features results in a distorted pentacoordinated sphere exhibiting a newly occupied coordination site, as confirmed by single crystal X-ray diffraction analysis. This particular geometry results in enhanced catalytic reactivity and is clearly reminiscent of the entatic state model. The proposed mechanism (Scheme 12) involves the insertion of a nitrene or carbene group to the copper complex **22** to form intermediate **23**. Subsequent alkene insertion yields species **25** and the group-transfer product (**21a**,**h–j**,**n**,**o**, **26a–g** and **27a–o**) is released upon ring-closure (Scheme 12). A wide range of substrates including diverse mono-, di-, and trisubstituted styrene derivatives (**21a**,**i**,**h**, **26a–d and 27a–i**) substrates as well as unactivated or deactivated tri- and tetrasubstituted scaffolds (**21j**,**n**,**o**, **26e–g** and **27j–o**) were efficiently converted.

## Conclusion

The use of redox-active ligands opens new venues in copper catalysis by utilizing the efficient electronic interplay between the metal center, the redox-active ligand and the reaction substrate. While such an behavior is a cornerstone in enzymatic reactions, the wide opportunities offered by these bioinspired approaches are only emerging and should blossom in the near future. In order to assert this field as a true game-changer in copper catalysis, further work should aim at rationalizing the “redox dialogue” between the metal and the ligand, and provide a deeper understanding of the valence tautomerism at work in such systems in order to develop predictive tools for reactivity control [41]. Such knowledge is most likely to result in new advances in the fast-expanding field of redox catalysis.
